# TSML: A New Pig Behavior Recognition Method Based on Two-Stream Mutual Learning Network

**DOI:** 10.3390/s23115092

**Published:** 2023-05-26

**Authors:** Wangli Hao, Kai Zhang, Li Zhang, Meng Han, Wangbao Hao, Fuzhong Li, Guoqiang Yang

**Affiliations:** 1 School of Software, Shanxi Agricultural University, Jinzhong 030801, China; haowangli@sxau.edu.cn (W.H.); rheal@stu.sxau.edu.cn (K.Z.); zxcvbn@stu.sxau.edu.cn (L.Z.); qwer0932@stu.sxau.edu.cn (M.H.); lifuzhong@sxau.edu.cn (F.L.); 2 Yuncheng National Jinnan Cattle Genetic Resources and Gene Protection Center, Yongji 044099, China; haowangbao@gmail.com

**Keywords:** pig breeding, behavior recognition, computer vision, two stream mutual learning, animal welfare

## Abstract

Changes in pig behavior are crucial information in the livestock breeding process, and automatic pig behavior recognition is a vital method for improving pig welfare. However, most methods for pig behavior recognition rely on human observation and deep learning. Human observation is often time-consuming and labor-intensive, while deep learning models with a large number of parameters can result in slow training times and low efficiency. To address these issues, this paper proposes a novel deep mutual learning enhanced two-stream pig behavior recognition approach. The proposed model consists of two mutual learning networks, which include the red–green–blue color model (RGB) and flow streams. Additionally, each branch contains two student networks that learn collaboratively to effectively achieve robust and rich appearance or motion features, ultimately leading to improved recognition performance of pig behaviors. Finally, the results of RGB and flow branches are weighted and fused to further improve the performance of pig behavior recognition. Experimental results demonstrate the effectiveness of the proposed model, which achieves state-of-the-art recognition performance with an accuracy of 96.52%, surpassing other models by 2.71%.

## 1. Introduction

Behavior changes play a crucial role in the pig breeding process. Accurately monitoring and understanding pig behavior is essential for improving pig welfare [1], predicting their health status, and facilitating the development of intelligent farming. To achieve promising pig behavior recognition performance, numerous researchers have conducted extensive studies. These studies can be broadly classified into two categories: sensor-based and computer vision-based approaches.

The first group of techniques relies on sensor-based monitoring of pig behavior. Several researchers have designed automatic monitoring systems that use sensors, such as infrared-sensitive cameras for real-time monitoring of pig activities [2] and behavior measurement. Other methods employ high-frequency radiofrequency identification (HF RFID) systems for monitoring individual drinking behavior [3] or pressure pads to track lame behavior in pigs [4]. However, these techniques involve physical contact with the pigs that can lead to stress and inaccurate measurements.

The second group of methods is based on computer vision. For instance, Zhang et al. [5] proposed a two-stream convolutional neural network for pig behavior recognition, where the feature extraction network is either a residual network (ResNet) or an inception network.

Zhuang et al. [6] developed a pig feeding and drinking behavior recognition model based on three models: VGG19, Xception, and MobileNetV2. They also designed two systems to monitor pig behaviors. Their final results demonstrated that the MobileNetV2-trained model had a significant advantage in pig behavior recognition, with a recall rate above 97%.

Wang et al. [7] implemented an improved HRNet-based method for joint point detection in pigs. By employing CenterNet to determine the posture of pigs (whether they are lying or standing), and then implementing the HRST approach for joint point detection in standing pigs, they achieved an average detection accuracy of 77.4%.

Luo et al. [8] proposed a channel-based attention model for real-time detection of pig posture. They compared their model with other popular network models, such as ResNet50, DarkNet53, and MobileNetV3, and showed that their proposed model outperformed the other models in terms of accuracy. They proved that the channel-based attention model is a promising approach for real-time pig posture detection [9].

Zhang et al. [10] presented an SBDA-DL, which is a deep learning-based real-time behavior-detection algorithm for sows. They designed it to detect three typical behaviors of sows: drinking, urinating, and sitting. The algorithm utilizes a combination of convolutional neural networks (CNN) and recurrent neural networks (RNN), along with a transfer learning approach, to achieve a high level of accuracy in behavior detection.

The experimental results showed that the average detection accuracy, measured by mean average precision (mAP), reached 93.4%, indicating the effectiveness of the proposed approach. The SBDA-DL algorithm provides a non-invasive method for monitoring sow behavior, which can reduce labor costs and enhance animal welfare in pig farming.

Li et al. [11] proposed a multi-behavioral spatio-temporal network model for pigs. By comparing it with a single-stream 3D convolutional model, the proposed model achieved a top-one accuracy of 97.63% on the test set. This multi-behavioral spatio-temporal network model provides a new approach for recognizing pig behaviors [12]. It has the potential to improve the efficiency of pig farming and to ensure animal welfare [13].

In summary, sensor-based methods are vulnerable to collision damage, resulting in inaccurate recognition and causing stress to the pigs both mentally and physically. Meanwhile, although deep-learning-based methods have achieved successful recognition results, their large parameter sizes lead to lengthy training and testing times, limiting their practical deployment on low-memory and low-capacity devices.

To overcome these challenges, we propose a novel two-stream mutual-learning (TSML) model for pig behavior recognition, aiming at improving the efficiency of pig farming and ensuring animal welfare. In comparison to other methods, TSML is more accurate and efficient in recognizing pig behavior. Our method is characterized by the cooperation between the RGB and flow streams that enables it to extract both appearance and temporal information efficiently. It also allows the model to extract critical feature information while avoiding irrelevant interference. Moreover, the mutual learning strategy improves the accuracy of behavior recognition by enabling the two student networks in each stream to learn collaboratively, gaining more robust and richer features in a shorter time. Compared with other methods that use either single-stream convolutional networks or multi-stream networks, our proposed model outperforms them in terms of accuracy, while being more efficient with a smaller number of parameters. This makes it more feasible to deploy on low-memory and low-capacity devices. Additionally, our unique dataset of pig behavior videos allows for more precise and reliable behavior detection and analysis, making our method practical for use in pig farming applications. Overall, our proposed two-stream mutual-learning method offers significant improvements over existing methods in terms of accuracy and efficiency while being practical for real-world applications.

The impact of our research on pig breeding is significant. Efficient monitoring of pig behavior is essential for improving pig welfare and for increasing the economic benefits of pig farms. Accurately monitoring and understanding pig behavior also allows for the prediction of their health status and facilitates the development of intelligent farming. The proposed TSML model offers a non-invasive and efficient method for monitoring pig behavior. In addition, by utilizing our unique dataset of pig behavior videos, future pig farming can be modernized with more precise and reliable behavior detection and analysis. Overall, our proposed method and dataset could significantly impact the pig breeding industry and enhance animal welfare.

Overall, the contributions of this paper can be summarised below:

- We established a novel dataset of pig behavior recognition dataset, which contains six categories. To provide a comprehensive understanding of pig behavior recognition, we have included six categories in our dataset, with each category consisting of roughly 600 videos. Each video varies in length from 5 to 10 s, providing sufficient footage to detect and analyze behavior patterns in pigs. These videos were collected over a period of one month utilizing six Hikvision cameras capturing over 85 pigs on a farm. All of the factors mentioned above have contributed to the creation of a unique and diverse dataset, collected on this farm, that exhibits better diversity in terms of illumination, angles, and other variables. This approach ensures that the dataset accurately represents the various scenarios and environments in which pigs behave, thereby resulting in more precise and reliable behavior detection and analysis.
- We first propose a novel pig behavior recognition method based on a two-stream mutual-learning framework. This model can efficiently extract more robust and richer features via mutual learning in RGB and flow paths separately and will extract both appearance and temporal information. Simultaneously, the decisions of the RGB and flow branches can be merged to gain improved pig behavior recognition performance. Specifically, our model achieves the best performance for pig behavior recognition task, with about a 2.71% improvement in the existing model.
- Several experiments were conducted to validate the superiority of the proposed model. The experiments included evaluating the performance of the proposed models, evaluating the behavior recognition performance of different models with or without mutual learning, evaluating the performance of the proposed model based on two identical networks, and evaluating the performance of the proposed model based on two different networks.

The rest of this paper can be organized as follows: Section 2 provides a detailed description of the methods and dataset used in the study. Section 3 presents the experimental results and analysis. In Section 4, we discuss the findings of our research. Finally, we conclude the paper in Section 5.

## 2. Materials and Methods

### 2.1. Datasets

**Figure 1:**
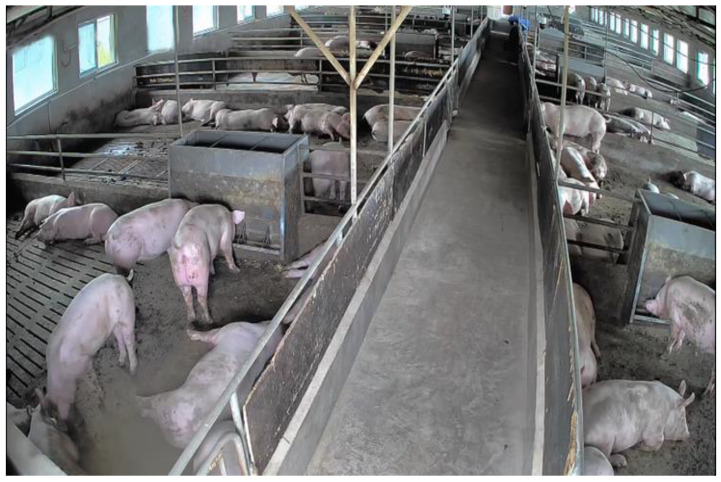
Environment of the pig farm.

The video data were collected from a pig farm located in Xiangfen County Agricultural Green Park Agricultural Company Limited, Linfen City, Shanxi Province. The farm encompasses 20 pig barns, each of which contains drinking water and feeding equipment as shown in Figure 1. For this study, six barns were selected, housing a total of 85 three-yuan fattening pigs. To ensure effective data collection, one camera was installed on each of the six barns at a height of approximately 3 m from the ground. The cameras were angled at 45 degrees diagonally toward the aisle and recorded videos at 25 fps with a resolution of 1920 × 1080 pixels. The specific camera utilized in this research was Hikvision DS-2DE3Q120MY-T/GLSE, and the whole data collection process lasted for 45 consecutive days, from 12 August 2022 to 25 September 2022.

**Figure 2:**
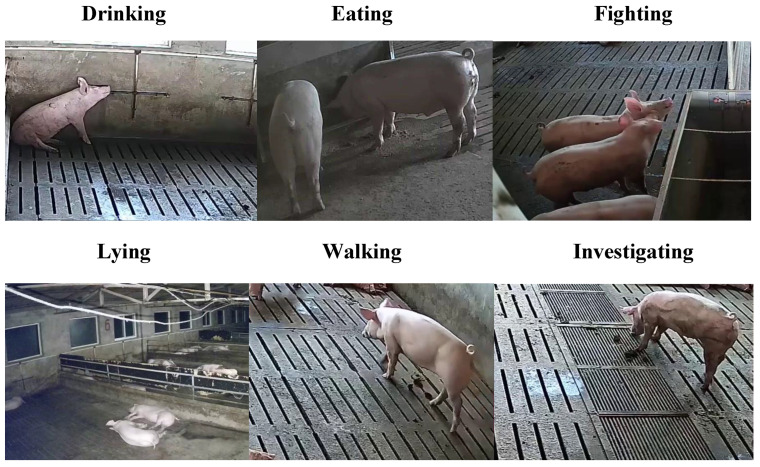
Sample of pig behavioural recognition dataset.

The final pig behavioral recognition dataset contains six categories, including fighting, drinking, eating [14], investigating, lying, and walking (as shown in Figure 2). Specifically, each category consists of approximately 600 videos, each lasting between 5 and 8 s. In total, the dataset contained 3606 videos, of which 80% (2886 samples) were utilized for training, and 20% (720 samples) were employed for testing. Further, the detailed distribution of the collected videos of different behavioral categories is shown in Table 1.

### 2.2. Problem Definition

This paper presents a novel TSML approach for pig behavior recognition. The model comprises two branches, spatial and temporal, each of which contains two student networks that perform mutual learning. The spatial branch extracts appearance features from still image frames while the temporal branch focuses on the optical flow motion in the video frames. The results of the two branches are subsequently weighted and fused to yield the final recognition result for pig behavior. The two-stream strategy employed in this approach effectively captures the complementary nature of the appearance and motion information underlying the video [15], while the mutual learning design further enhances the efficiency and accuracy of the model in recognizing pig behavior [16].

**Figure 3:**
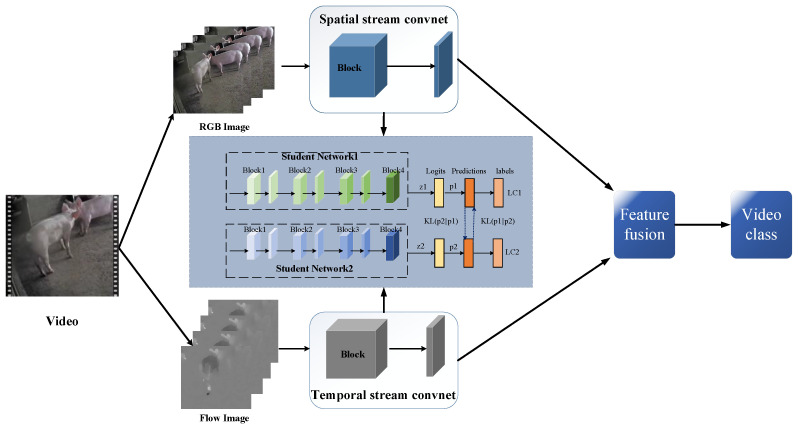
The structure diagram of the two-stream network model based on the idea of mutual learning.

The framework of the proposed TSML is presented in Figure 3. The input to the framework are *M* videos $V={\left\{v_{i}\right\}}_{i=1}^{M}$ from *C* classes, with the corresponding video behavior label set denoted as $Y={\left\{y_{i}\right\}}_{i=1}^{M}$, where $y_{i}\in \left\{1,2,\cdots ,C\right\}$.

The probability, $p_{s1}^{c}\left(x_{i}^{s}\right)$, of the RGB image $x_{i}^{s}$ from the ith video $v_{i}$ belonging to class *c* in the first student network of the spatial stream can be calculated as follows:

(1) $$p_{s1}^{c}\left(x_{i}^{s}\right)=\frac{exp(S_{s1}^{c}\left(x_{i}^{s}\right))}{\sum_{c=1}^{C}exp\left(S_{s1}^{c}\left(x_{i}^{s}\right)\right)}$$

Here, $S_{s1}^{c}\left(x_{i}^{s}\right)$ represents the logit output of the softmax layer from the first student network in the spatial stream for input $x_{i}^{s}$.

The probability, $p_{s2}^{c}\left(x_{i}^{s}\right)$, of the RGB image $x_{i}^{s}$ from the ith video $v_{i}$ belonging to class *c* in the second student network of the spatial stream can be written as follows:

(2) $$p_{s2}^{c}\left(x_{i}^{s}\right)=\frac{exp(S_{s2}^{c}\left(x_{i}^{s}\right))}{\sum_{c=1}^{C}exp\left(S_{s2}^{c}\left(x_{i}^{s}\right)\right)}$$

Similarly, $S_{s2}^{c}\left(x_{i}^{s}\right)$ represents the logit output of the softmax layer from the second student network in the spatial stream for input $x_{i}^{s}$.

The probability, $p_{t1}^{c}\left(x_{i}^{t}\right)$, of flow image $x_{i}^{t}$ corresponding to the RGB image $x_{i}^{s}$ from the ith video $v_{i}$ belonging to class *c* in the first student network of the temporal stream can be described as follows:

(3) $$p_{t1}^{c}\left(x_{i}^{t}\right)=\frac{exp(S_{t1}^{c}\left(x_{i}^{t}\right))}{\sum_{c=1}^{C}exp\left(S_{t1}^{c}\left(x_{i}^{t}\right)\right)}$$

On the other hand, $S_{t1}^{c}\left(x_{i}^{t}\right)$ denotes the logit output of the softmax layer from the first student network in the flow stream for input $x_{i}^{t}$.

The probability, $p_{t2}^{c}\left(x_{i}^{t}\right)$, of flow image $x_{i}^{t}$ corresponding to the RGB image $x_{i}^{s}$ from the ith video $v_{i}$ belonging to class *c* in the second student network of the temporal stream can be calculated as follows:

(4) $$p_{t2}^{c}\left(x_{i}^{t}\right)=\frac{exp(S_{t2}^{c}\left(x_{i}^{t}\right))}{\sum_{c=1}^{C}exp\left(S_{t2}^{c}\left(x_{i}^{t}\right)\right)}$$

Similarly, $S_{t2}^{c}\left(x_{i}^{t}\right)$ represents the logit output of the softmax layer from the second student network in the flow stream for input $x_{i}^{t}$.

The loss functions for the spatial and temporal two branches in the TSML can be defined as:

(5) $$\begin{array}{c} L_{s}=\left(1-\alpha \right)\times L_{s1}+\alpha \times L_{s2}\\ L_{t}=\left(1-\alpha \right)\times L_{t1}+\alpha \times L_{t2} \end{array}$$

Here, $L_{s}$ and $L_{t}$ represent the loss of the spatial stream and the temporal stream, respectively. The hyperparameter α controls the balance between these two loss terms. Furthermore, $L_{s1}$ and $L_{s2}$ denote the losses for the two student networks in the spatial stream, while $L_{t1}$ and $L_{t2}$ denote the losses for the two student networks in the temporal stream.

The formulations for $L_{s1}$ and $L_{t1}$ are as follows:

(6) $$\begin{array}{c} L_{s1}=L_{c\_s1}+D_{KL}(p_{s1}\left|\right|p_{s2})\\ L_{t1}=L_{c\_t1}+D_{KL}(p_{t1}\left|\right|p_{t2}) \end{array}$$

Here, $L_{c\_s1}$ and $L_{c\_t1}$ represent the cross-entropy loss that measures the difference between the predicted value and the actual value. $D_{KL}(p_{s1}\left|\right|p_{s2})$ represents the Kullback–Leibler (KL) divergence between the probability distributions $p_{s1}$ and $p_{s2}$. $L_{c\_s1}$ and $L_{c\_t1}$ can be calculated using the following equation:

(7) $$\begin{array}{c} L_{c\_s1}=-\sum_{i=1}^{M}\sum_{c=1}^{C}y_{i}^{c}log\left(p_{s1}^{c}\left(x_{i}^{r}\right)\right)\\ L_{c\_t1}=-\sum_{i=1}^{M}\sum_{c=1}^{C}y_{i}^{c}log\left(p_{t1}^{c}\left(x_{i}^{t}\right)\right) \end{array}$$

Among them, $y_{i}^{c}$ is an indicator, if $y_{i}=c$, $y_{i}^{c}=1$; and if $y_{i}\neq c$, $y_{i}^{c}=0$.

In the spatial stream, to enhance the generalization capacity of the first student network on testing samples, another peer network is employed to provide training experience via its posterior probability p2. The KL divergence is used to quantify the matching degree between the predictions p1 and p2. $D_{KL}(p_{s2}\left|\right|p_{s1})$ indicates the KL distance from $p_{s1}$ to $p_{s2}$ and can be calculated using the following formula:

(8) $$\begin{array}{c} D_{KL}\left(p_{s2}\left|\right|p_{s1}\right)=\sum_{i=1}^{M}\sum_{c=1}^{C}p_{s2}^{c}\left(x_{i}^{r}\right)log\frac{p_{s2}^{c}\left(x_{i}^{r}\right)}{p_{s1}^{c}\left(x_{i}^{r}\right)} \end{array}$$

Here, $p_{s1}$ and $p_{s2}$ represent the predicted probability distributions from the first and second student networks, respectively, in the spatial stream.

In the temporal stream, $D_{KL}(p_{t2}\left|\right|p_{t1})$ indicates the KL distance from $p_{t1}$ to $p_{t2}$ and shares a similar meaning with $D_{KL}(p_{s2}\left|\right|p_{s1})$ in the spatial stream.

Moreover, the meanings of $L_{s2}$ and $L_{t2}$ are similar to those of $L_{s1}$ and $L_{t1}$.

### 2.3. The Implementation Details

The software and hardware system settings used in this paper are presented in Table 2. For fair comparison, we optimized all experimental models with a gradient descent algorithm using a momentum of 0.9, a batch size of 16, a learning rate of 0.001, and an Alpha value of 0.5, and we trained them for 500 epochs.

### 2.4. Evaluation Criteria

In order to compare the performance of different models, several evaluation criteria were used, including accuracy, parameters, FLOPs (floating point operations per second), and loss. The accuracy reflects how well the model performs, while the number of parameters indicates the efficiency of the model—a smaller number of parameters is generally better. The FLOPs metric also indicates efficiency—again, a smaller number is better. It specifies the number of floating point operations required per second. All experiments were conducted on TITANX GPUs.

## 3. Experimental Results and Analysis

In this section, we will provide a detailed report on the experimental results and analysis. The overall experiment consists of several design parts, including evaluating the superiority of the proposed model, evaluating the efficiency of two stream mutual learning based on two identical networks, and evaluating the efficiency of two-stream mutual learning based on two different networks.

### 3.1. Evaluating the Superiority of the Proposed Model

To validate the superiority of the proposed TSML, several models were utilized for comparison, including ResNet18, ResNet34, ResNet50, Vgg16 [17] and MobileNetv2 [18]. The results are shown in Table 3.

Table 3 shows that the proposed model outperforms other common models in pig behavior recognition. Specifically, the proposed model achieves 96.52% accuracy, which is 4.51%, 0.87%, 2.19%, 2.18% better than the accuracy rates of ResNet18 [19], ResNet50 [19], MobileNetV2, VGG16, respectively. These results demonstrate the superiority of the proposed model.

**Figure 4:**
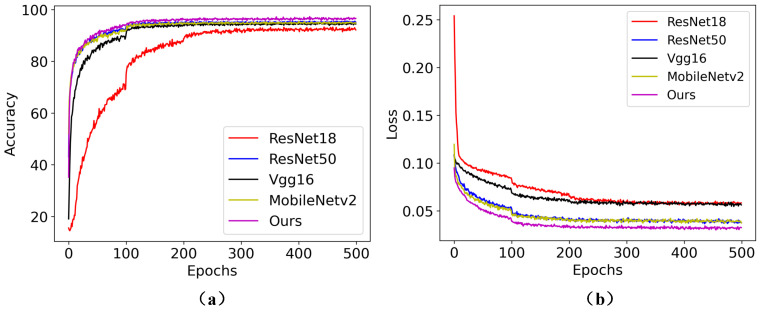
Comparison accuracies and losses of different models under different epochs for pig behavior recognition. (**a**) Accuracy for different models under different epochs. (**b**) indicates the Loss of different models under different epochs.

Furthermore, to provide readers with a more intuitive understanding of the superiority of TSML in pig behavior recognition, we report the accuracies and losses of different comparison models under different epochs in Figure 4. Here, Figure 4a shows the accuracy of different models under different epochs, while Figure 4b shows the loss of different models under different epochs.

The results in Figure 4 demonstrate that the accuracy and loss of TSML exceed those of other models, which further validates the effectiveness of the proposed TSML.

The outstanding performance of the TSML model can be attributed to its ability to effectively capture richer appearance and motion features [20], resulting in improved accuracy in pig behavior recognition tasks [21].

### 3.2. Evaluating the Efficiency of the Two-Stream Network in Pig Behavior Recognition

To validate the effectiveness of the two-stream network setting in the pig behavior [22] recognition framework, we compared the single RGB stream, single flow stream, and the fusion of two streams for several models [23], including ResNet18, ResNet34, ResNet50, Vgg16, MobileNetv2. The results of the comparison are displayed in Table 4.

Table 4 clearly demonstrates that the two-stream network setting consistently outperforms the single RGB and the flow networks by a significant margin.

To be more specific, the two-stream version of the ResNet18 model achieved an accuracy of 92.35%, which is 1.22% and 50.23% higher than its corresponding RGB and flow versions, respectively. The two-stream version of the ResNet50 model achieved an accuracy of 95.69%, which is 2.70% and 10.79% better than its corresponding RGB and flow versions, respectively. The two-stream version of the MobileNetv2 model achieved an accuracy of 94.45%, which is 0.60% and 9.52% better than its corresponding RGB and flow streams. The two-stream version of the Vgg16 model achieved an accuracy of 94.44%, which is 0.45% and 13.36% better than its corresponding RGB and flow versions, respectively.

**Figure 5:**
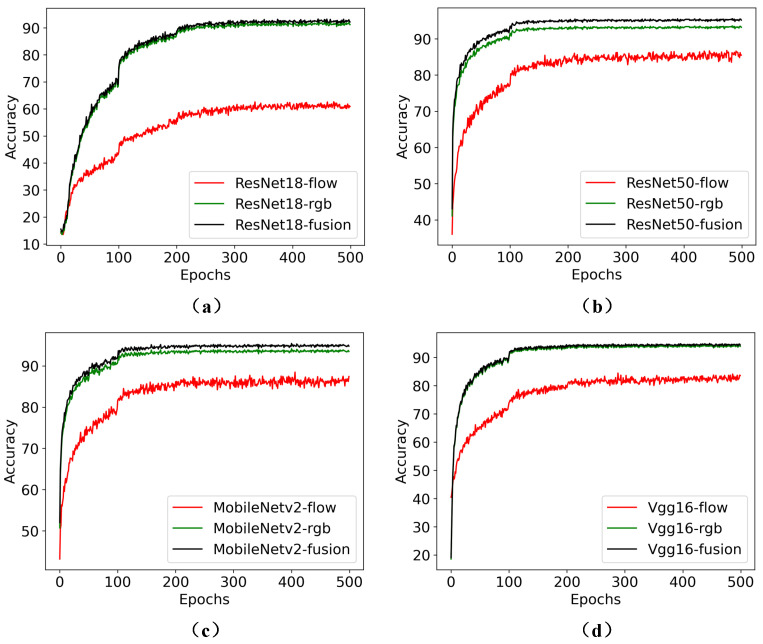
Comparison accuracies of RGB stream, flow stream and fusion of two streams based on different basic networks. (**a**) Comparison results based on ResNet18. (**b**) Comparison results based on ResNet50. (**c**) Comparison results based on MobileNetv2. (**d**) Comparison results based on Vgg16.

Furthermore, to provide readers with a more intuitive understanding of the two-stream network, we include Figure 5. These figures illustrate the accuracies and losses of the RGB, flow, and two stream settings of different basic models at various epochs. Here, of Figure 5a denotes the comparison results based on ResNet18; Figure 5b indicates the comparison results based on ResNet50; Figure 5c represents the comparison results based on MobileNetv2; Figure 5d is the comparison results based on Vgg16.

As depicted in Figure 5, the fusion of RGB and flow into two streams consistently achieved better results compared to using the RGB or flow streams alone. These results clearly demonstrate the superiority of the two-stream settings in the pig behavior recognition task. The use of both streams provides complementary information, allowing for more accurate and robust recognition of pig behaviors [24]. The fusion of multiple modalities has been a popular trend in many computer vision tasks, and our results provide evidence supporting this trend in the field of pig behavior recognition.

The results of these comparisons provide evidence of the superiority of the two-stream network in the pig behavior recognition task. The reason for this is that the two-stream network is capable of capturing both the appearance and motion information in the video, so that effective spatiotemporal features can be extracted, ultimately facilitating improved performance in pig behavior recognition. The RGB stream is capable of capturing appearance features such as color and texture, while the flow stream focuses on motion features such as the intensity and direction of movement. By combining both streams, our proposed two-stream network can effectively capture the complex spatiotemporal information for more precise and reliable recognition of pig behavior. Compared with traditional single-stream convolutional networks [25], using two streams allows for more efficient extraction of information. This approach reduces noise and irrelevant information while improving the accuracy of the recognition process. As a result, our proposed two-stream network provides a practical and viable approach for reliable pig behavior recognition in real-world applications.

In summary, the two-stream network is considered superior for pig behavior recognition tasks due to its ability to capture both appearance and motion information effectively. By processing this information jointly, our TSML model can generate more robust and accurate feature representation, making it a promising choice for pig behavior recognition.

### 3.3. Evaluating the Efficiency of TSML Based on Two Identical Networks

In this section, we evaluate the performance of our proposed TSML approach based on two identical student networks. Specifically, TSML utilized different backbone architectures, including ResNet18, ResNet50, MobileNetV2, to validate the generalization of the proposed approach. To simplify the explanation, we refer to these models as Res18, Res50, and Mobilev2, respectively. The comparison results are shown in Table 5. Among Table 5, SigRes18 refers to the RGB and flow two-stream networks that comprise a single Res18 network. MulRes18(Res18) indicates that both the RGB and flow networks consist of two student networks that perform mutual learning, with each branch of the student network based on the Res18 architecture. Other single models (SigRes50 and SigMobv2) and other mutual models (MulRes18, MulRes50 and MulMobv2) share similar meanings with those of Sig18 and MulRes18 (Res18). Furthermore, MulRes18(18)-i, denotes the index of two mutual-learning [26] models.

Table 5 illustrates that the TSML with two identical networks achieves significantly and consistently superior performance than those of the single network. Specifically, MulRes18(Res18)-1/MulRes18(Res18)-2 obtain 2.26%/2.41% better accuracy than that of sigRes18; MulRes50(Res50)-1/MulRes50(Res50)-2 obtain 0.86%/0.57% better accuracy than that of sigRes50; and MulMobv2(Mobilev2)-1/MulMobilev2(Mobilev2)-2 obtain 0.29%/0.15% better accuracy than that of SigMobilev2. These results validate the superiority of the TSML approach, which is based on two identical student networks for both the RGB and optical flow branches.

**Figure 6:**
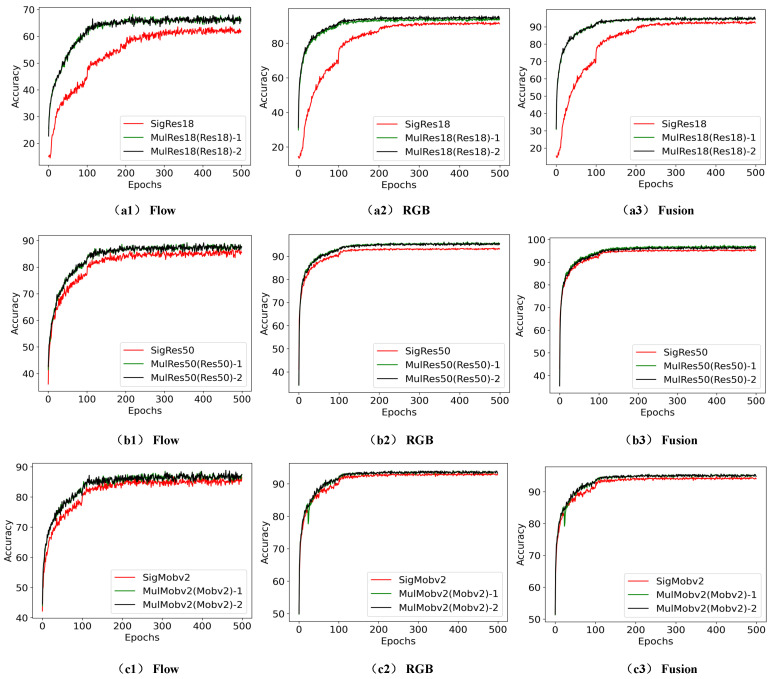
Comparison between accurate values of pig behaviour recognition based on mutual learning models of the two same networks. (**a1**–**a3**) represents the accuracy of the RGB/flow/fusion stream on the SigRes18 and MulRes18(Res18) models under different epochs. (**b1**–**b3**) presents the accuracy of the RGB/flow/fusion stream on the SigRes50 and MulRes50(Res50) models under different epochs. (**c1**–**c3**) denotes the accuracy of the RGB/flow/fusion stream on the SigMobilev2 and MulMobilev2(Mobilev2) models under different epochs.

**Figure 7:**
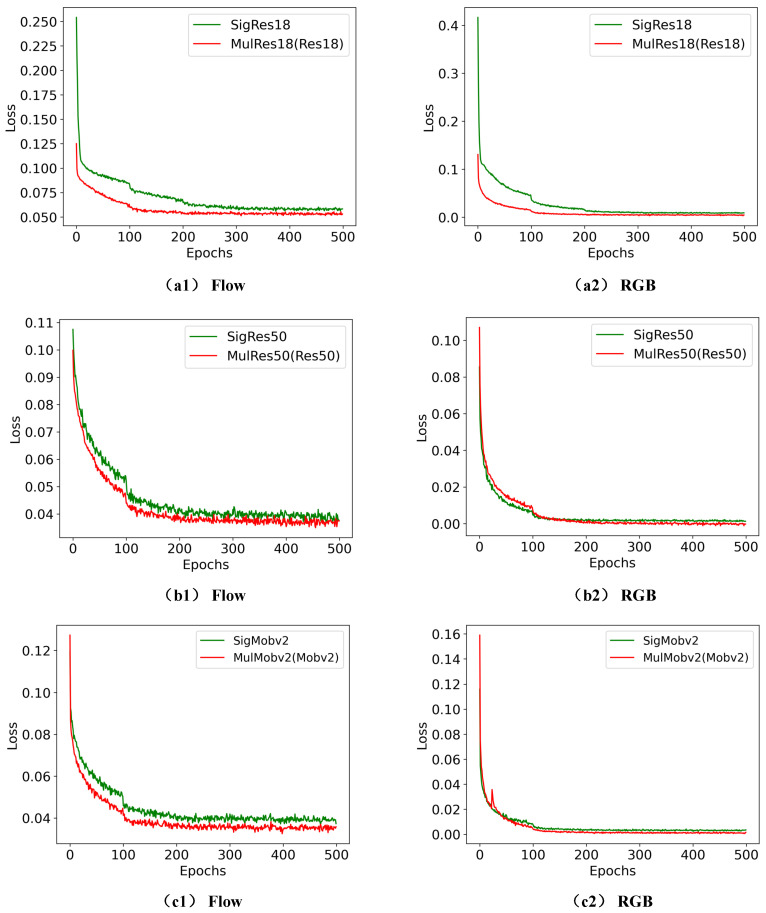
Comparison of loss values for pig behaviour recognition in mutual-learning models based on the two same networks. (**a1**,**a2**) represents the loss of the RGB/flow/fusion stream on the SigRes18 and MulRes18(Res18) models under different epochs. (**b1**,**b2**) presents the Loss of the RGB/flow/fusion stream on the SigRes50 and MulRes50(Res50) models under different epochs. (**c1**,**c2**) denotes the Loss of the RGB/flow/fusion stream on the SigMobilev2 and MulMobilev2(Mobilev2) models under different epochs.

Additionally, in order to provide readers with a more intuitive understanding and visualization of the superiority of TSML based on two identical networks, we include Figure 6 and Figure 7 that show the accuracies and losses of the different comparison models with and without mutual learning at various epochs.

Specifically, (a1)/(a2)/(a3) of Figure 6 represent the accuracy of the RGB/flow/fusion stream on the SigRes18 and MulRes18(Res18) models under different epochs; (b1)/(b2)/(b3) of Figure 6 present the accuracy of the RGB/flow/fusion stream on the SigRes50 and MulRes50(Res50) models under different epochs.

Furthermore, (a1)/(a2) of Figure 7 represent the loss of the RGB/flow/fusion stream on the SigRes18 and MulRes18(Res18) models under different epochs; (b1)/(b2) of Figure 7 present the Loss of the RGB/flow/fusion stream on the SigRes50 and MulRes50(Res50) models under different epochs. These figures provide useful insights into the performance of each stream on different backbone networks and how they evolve over time.

Figure 6 and Figure 7 demonstrate that the accuracy and the loss of MulRes18(Res18) and MulMobilev2(Mobilev2) outperform that of SigRes18 and SigMobilev2, which validates the effectiveness of the TSML based on two identical student networks.

The reason why the TSML model based on two identical student networks achieves better performance is as follows. Although the two student networks in the TSML model have the same network structure, their initial parameter values differ, resulting in the acquisition of different knowledge. Therefore, during the training process, they can obtain diverse knowledge and experience from each other, leading to the model producing better and more efficient behavior recognition performance.

### 3.4. Evaluating the Efficiency of TSML Based on Two Different Networks

In this section, we evaluate the performance of the proposed TSML approach using two different student networks.TSML utilized different backbone architectures, including ResNet18, ResNet34, and ResNet50. For ease of reference, we will refer to these models as Res18, Res34, Res50, and Mobilev2. The comparison results are shown in Table 6. In Table 6, the SigRes18 model refers to both the RGB and optical flow streams of TSML comprising a single Res18 network. Other single models share similar meanings as SigRes18. MulRes18(Res34) and MulRes34(Res18) indicate the two different mutual-learning student networks in the two streams of TSML that share the same structure with that of ResNet18 and ResNet34. Other mutual-learning models, such as MulRes18(Res50)/MulRes50(Res18) and MulRes34(Res50)/MulRes50(Res34), share similar meanings as MulRes18(Res34)/MulRes34(Res18).

Table 6 demonstrates that TSML with two different networks consistently achieves significantly superior performance compared to the corresponding single networks. Specifically, MulRes18(Res34)/MulRes34(Res18) achieve 2.41%/1.61% better accuracy than SigRes18/SigRes34; MulRes18(Res50)/MulRes50(Res18) demonstrate 2.71%/0.52% superior accuracy than SigRes18/SigRes34; and MulRes34(Res50)/MulRes50(Res34) achieve 1.77%/0.72% better accuracy than SigRes18/SigRes34. These results highlight the superiority of the TSML approach that employs two different student networks for both the RGB and optical flow branches.

In some cases, smaller student networks with mutual learning can outperform larger single neural networks.

The above experimental results indicate that the TSML model based on different student networks has superiority. This is attributed to the fact that in this model, two student networks have different network structures and initial parameter values, resulting in different knowledge. Consequently, their collaborative learning allows them to obtain different knowledge and experience from their peers, thereby achieving superior performance.

## 4. Discussions

The proposed TSML approach leverages both the mutual-learning and two-stream network strategies to gather enhanced appearance and motion information underlying video in an interactive manner. The cooperation between the RGB and flow streams enables the TSML to achieve promising accuracy and efficiency. The mutual-learning strategy allows the two student networks in each stream to learn collaboratively, gaining more robust and richer features in a shorter time, which further enhances the accuracy of pig behavior recognition. Our approach not only improves the accuracy of pig behavior recognition, but it also enhances the efficiency of the recognition process. To validate the superiority of TSML, several experiments were designed and conducted, including evaluation of the superiority of the TSML model and evaluation of the TSML model based on two of the same or different student networks.

The experiments demonstrated that our proposed TSML model outperforms other models for pig behavior recognition, achieving an improvement of about 2.71% in accuracy. Specifically, the TSML model achieved 96.52% accuracy, which is 4.51%, 0.87%, 2.19%, 2.18% better than those of ResNet18, ResNet50, MobileNetV2, and Vgg16, respectively. To sum up, the experimental results demonstrate that our TSML model outperforms the competition in terms of accuracy when applied to the pig behavior recognition task.

The outstanding performance of the TSML model can be attributed to its ability to effectively capture richer appearance and motion features. By leveraging the two-stream mutual-learning framework, the model can efficiently extract both appearance and temporal information, leading to enhanced feature representation and improved accuracy in pig behavior recognition tasks. The RGB stream captures appearance features such as color and texture, while the flow stream captures motion features such as the intensity and direction of movement. By combining both streams and by collaboratively learning between them, our TSML model is better able to capture the complex visual cues that are critical for pig behavior recognition. In contrast to other approaches, our TSML model is specifically designed to balance the performance and efficiency trade-off in pig behavior recognition tasks. By utilizing mutual-learning and two-stream network strategies, the model can capture more robust features with fewer parameters, making it more practical for real-world applications. This approach provides a comprehensive understanding of pig behavior and further insights on the creation of a robust deep network that can be applied to various tasks.

Furthermore, our experimental results demonstrate that the TSML model with two different or same networks in both the RGB and flow streams consistently achieves significantly superior performance compared to their corresponding single network. This improvement can be attributed to several factors. Firstly, by using two student networks with unique initial parameter values or network structures, the TSML model can gain different knowledge and acquire a more comprehensive understanding of the appearance or flow of information in the videos. This approach allows the networks to learn from each other, leading to a more robust and comprehensive feature representation that enhances the accuracy of pig behavior recognition. Additionally, the collaborative learning of the student networks allows them to acquire different knowledge and experience from their peers. This approach enhances their ability to recognize pig behavior more accurately and efficiently. By combining these mechanisms, our proposed model achieves a high level of performance in pig behavior recognition. In summary, our experimental results suggest that using multiple student networks within the TSML model can significantly improve pig behavior recognition accuracy and efficiency. The benefit of mutual learning and information fusion between different networks provides a substantial gain that can be performance-driven in various domains.

However, one potential disadvantage of our TSML model is that it requires a larger amount of training data to achieve optimal performance. Nonetheless, given the significant improvement in accuracy, this method is considered suitable for practical applications in pig farming.

To further improve the accuracy and efficiency of the model, future work could explore the use of other advanced machine learning techniques such as reinforcement learning, transfer learning, and attention mechanisms. Additionally, future studies could apply our proposed approach to other domains such as wildlife conservation for animal behavior recognition.

## 5. Conclusions

This paper proposes a novel approach for pig behavior recognition, named TSML, which combines mutual learning with two stream neural networks that separately learn both appearance and motion information from videos. The mutual-learning strategy ensures that the basic student neural networks in the model update parameters collaboratively and gain information from each other throughout the training process. Furthermore, the two-stream network collects both appearance and motion information via its RGB and flow branches. Leveraging mutual learning and the two-stream network, the TSML model achieves excellent pig behavior recognition performance with higher efficiency and effectiveness. The experimental results show that the TSML model can greatly improve pig behavior recognition performance, delivering 2.71% higher accuracy in comparison to other models.

In terms of future work, we will explore the application of the proposed model to behavior recognition tasks for other livestock such as cattle and sheep. Additionally, we will continue to investigate more efficient and effective network structures to enhance the accuracy and efficiency of pig behavior recognition. Lastly, we will explore effective methods for identifying complex group pig behaviors.

## Figures and Tables

**Table 1:** Number of videos of different pig behaviours.

Behavior	Number
Fighting	605
Drinking	597
Eating	607
Investigating	602
Lying	601
Walking	594
Total	3606

**Table 2:** Experimental environment configuration information.

Categories	Type or Version
Operating system	Ubuntu 18.04.5 LTS 64-bite
CPU	Intel Core i7-7800X @ 3.5 GHz*12
GPU	NVIDIA TITAN Xp
Memory	128 GB
Hard Disk	4TB SSD*3
Python	3.6.9
Pytorch	1.2.0
CUDA	11.2
CUDNN	10.0.130

**Table 3:** Comparison of different network models for pig behaviour recognition.

Model	Accuracy (%)
ResNet18	92.35
ResNet50	95.69
MobileNetv2	94.45
Vgg16	94.44
Ours	96.52

**Table 4:** Comparison of pig behavior recognition accuracy based on two mutual learning models of the same network.

Model	Flow (%)	RGB (%)	Two-Stream Fusion (%)
ResNet18	61.47	91.24	92.35
ResNet50	86.37	93.18	95.69
MobileNetv2	86.23	93.88	94.45
Vgg16	83.31	94.02	94.44

**Table 5:** Comparison of pig behavior recognition accuracy based on two mutual-learning models of the same network.

Model	Flow (%)	RGB (%)	Two-Stream Fusion (%)
SigRes18	61.47	91.24	92.35
MulRes18(Res18)-1	66.20	93.60	94.44
MulRes18(Res18)-2	66.62	94.02	94.58
SigRes50	86.37	93.18	95.69
MulRes50(Res50)-1	87.67	95.97	96.52
MulRes50(Res50)-2	87.26	95.41	96.24
SigMobv2	86.23	93.88	94.45
MulMobv2(Mobilev2)-1	87.02	93.32	94.71
MulMobv2(Mobilev2)-2	87.07	92.49	94.58

**Table 6:** Comparison of the accuracy of pig behaviour recognition based on mutual-learning models of two different networks, ResNet18 and ResNet34.

Model	Flow (%)	RGB (%)	Two-Stream Fusion (%)
SigRes18	61.47	91.24	92.35
SigRes34	65.23	93.74	94.16
MulRes18(Res34)	64.67	94.44	94.58
MulRes34(Res18)	67.87	94.99	95.68
SigRes18	61.47	91.24	92.35
SigRes50	86.37	93.18	95.69
MulRes18(Res50)	71.22	94.71	94.85
MulRes50(Res18)	87.36	95.55	96.19
SigRes34	65.23	93.74	94.16
SigRes50	86.37	93.18	95.69
MulRes34(Res50)	72.34	95.55	95.83
MulRes50(Res34)	88.63	95.69	96.38

## Data Availability

The datasets generated and/or analyzed during the current study are available from the corresponding author on reasonable request.
